# Desorption of Ammonia Adsorbed on Prussian Blue Analogs by Washing with Saturated Ammonium Hydrogen Carbonate Solution

**DOI:** 10.3390/molecules27248840

**Published:** 2022-12-13

**Authors:** Hatsuho Usuda, Yoshie Mishima, Tohru Kawamoto, Kimitaka Minami

**Affiliations:** Nanomaterials Research Institute, National Institute of Advanced Industrial Science and Technology (AIST), 1-1-1 Higashi, Tsukuba 305-8565, Japan

**Keywords:** Prussian blue analogs, ammonia, ammonium hydrogen carbonate, adsorption

## Abstract

Prussian blue analogs (PBAs) have been reported as promising ammonia (NH_3_) adsorbents with a high capacity compared to activated carbon, zeolite, and ion exchange resins. The adsorbed NH_3_ was desorbed by heating and washing with water or acid. Recently, we demonstrated that desorption was also possible by washing with a saturated ammonium hydrogen carbonate solution (sat. NH_4_HCO_3_ *aq*) and recovered NH_3_ as an NH_4_HCO_3_ solid by introducing CO_2_ into the washing liquid after desorption. However, this has only been proven for copper ferrocyanide and the relationship between the adsorption/desorption behavior and metal ions in PBAs has not been identified. In this study, we investigated the adsorption/desorption behavior of PBAs that are complexes of first row transition metals with hexacyanometalate anions. Six types of PBAs were tested in this study and copper ferricyanide exhibited the highest desorption/adsorption ratio. X-ray diffraction results revealed high structural stability for cobalt hexacyanocobaltate (CoHCC) and nickel ferricyanide (NiHCF). The Fourier transform infrared spectroscopy results showed that the NH_3_ adsorbed on the vacancy sites tended to desorb compared to the NH_3_ adsorbed on the interstitial sites as ammonium ions. Interestingly, the desorption/adsorption ratio exhibited the Irving-Williams order.

## 1. Introduction

An active nitrogen compound, ammonia (NH_3_), is widely used as raw material for fertilizers, while its emission into the environment has caused various problems such as eutrophication, acidification, and aerosols (PM2.5) [1,2]. The production of NH_3_ was estimated to be 182 million metric tons in 2021 [3], most of which was used as a synthetic fertilizer in farms for efficient plant growth [4]. The main production method of NH_3_, the Haber-Bosch process, requires a temperature of 400–600 °C and pressure of 100–200 bar. NH_3_ emissions mainly occur in agriculture owing to manure management, evaporation from fertilizers, and grazing. Indeed, 94% of the total NH_3_ emissions were from agriculture in 2016 [5]. Bio-filters and activated sludge processes, which use bacteria to decompose ammonia into atmospheric nitrogen (N_2_) usually conduct the detoxification of ammonia. These technologies consume a lot of energy on aeration for biological treatments, only for converting NH_3_ back to useless N_2_ [6]. Besides, these detoxification techniques do not treat all the NH_3_ emitted from agriculture and industry, resulting in the emission of 70 million tons of NH_3_ per year into the atmosphere [7]. These facts show that if we recycle ammonia, we will solve both pollution and energy issues.

To recycle NH_3_, novel techniques are needed to collect it and convert it into a reusable form. Existing methods for collecting gaseous NH_3_ mainly involve water scrubbing and adsorption [8,9,10,11,12]. Water scrubbing takes up space, requires a certain amount of energy for temperature control, and yields a large amount of wastewater. Adsorbents can downsize the NH_3_ processing system when they have high adsorption capacity.

**Figure 1:**
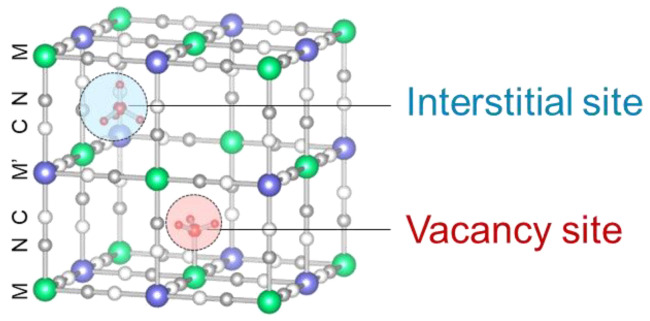
The crystal structure of PBA with a hexacyanoferrate vacancy at the center. The interstitial sites and vacancy sites are exhibited as red and blue balls, respectively.

Prussian blue (PB) and Prussian blue analogs (PBAs) have been reported to adsorb ammonia selectively and exhibit higher adsorption capacities than other ammonia adsorbents [11,13,14]. PB is a mesoporous material that is often used as an adsorbent [15,16,17] and is represented as A*_y_*Fe^III^[Fe^II^(CN)_6_]*_x_*, A = Na^+^, K^+^, NH_4_^+^, etc. It has been well investigated since it was used as a pigment in the 18th century [18]. Fe^3+^ and Fe^2+^ in A*_y_*Fe^III^[Fe^II^(CN)_6_]*_x_* can be substituted into other metals, such as cobalt, copper, and manganese, to yield PBAs exhibiting different adsorption and desorption properties and stabilities [13,19,20,21,22]. There are two types of adsorption sites in PBAs as shown in Figure 1 [13]. One is an interstitial site, which is a confined space where a positive ion is preferentially adsorbed and the other is a vacancy site surrounded by six open metal sites where NH_3_ and water (H_2_O) can be adsorbed. Another outstanding property of PBAs is that the desorption of adsorbed NH_3_ can regenerate them via heating or washing with solutions [13,23,24,25].

If NH_3_ was desorbed from the PBAs even into a saturated ammonium hydrogen carbonate solution (sat. NH_4_HCO_3_ *aq*), NH_3_ can be converted to solid ammonium hydrogen carbonate (NH_4_HCO_3_). The system uses the characteristic property that NH_3_ can dissolve even in sat. NH_4_HCO_3_ *aq*. This solution with excess dissolved NH_3_ yields solid NH_4_HCO_3_ when carbon dioxide (CO_2_) is introduced into the solution because the chemical composition of the system falls within the range of phase separation [26,27]. Recently, our group reported NH_3_ adsorbed on Cu^II^[Fe^II^(CN)_6_]_0.5_ could be desorbed into sat. NH_4_HCO_3_ *aq* [23], and the desorbed NH_3_ converted into solid NH_4_HCO_3_ by blowing CO_2_ into the solution. However, NH_3_ desorption into sat. NH_4_HCO_3_ *aq* has been proven only for Cu^II^[Fe^II^(CN)_6_]_0.5_, and the relationship between the adsorption/desorption behavior and metal ions in PBAs is not clear. In the case of Cu^II^[Fe^II^(CN)_6_]_0.5_, NH_3_ is adsorbed as NH_3_ molecules on both the vacancy sites and interstitial sites and as ammonium ions on the interstitial sites. The structure of Cu^II^[Fe^II^(CN)_6_]_0.5_ changed upon adsorption and remained unchanged upon desorption. Other PBAs can have more effective NH_3_ adsorption/desorption behavior for NH_3_ recovery as NH_4_HCO_3_ solids than Cu^II^[Fe^II^(CN)_6_]_0.5_. The relationship between the adsorption/desorption behavior and the metal ions in PBAs needs to be clarified to find a PBA with more effective adsorption/desorption characteristics for practical use.

In this study, we investigated the adsorption/desorption behavior of PBAs that are complexes of first row transition metals with hexacyanometalate anions. We tested six types of PBAs: copper hexacyanoferrate (CuHCF), cobalt hexacyanocobaltate (CoHCC), nickel hexacyanoferrate (NiHCF), zinc hexacyanoferrate (ZnHCF), cobalt hexacyanoferrate (CoHCF), and manganese hexacyanoferrate (MnHCF). CuHCF exhibited the highest desorption amount and ratio. Fourier transform infrared spectroscopy (FTIR) showed that the ammonia molecules adsorbed on the vacancy sites tended to desorb compared to the ammonia adsorbed on the interstitial sites as ammonium ions. The desorption/adsorption ratio exhibited the same tendency as that of the Irving-Williams series. The X-ray diffraction (XRD) results showed that structural stability was high for cobalt hexacyanocobaltate (CoHCC) and nickel ferricyanide (NiHCF).

## 2. Results and Discussion

### 2.1. The Amount of NH_3_ Adsorbed and Desorbed

The amount of NH_3_ adsorbed from gas and desorbed into sat. NH_4_HCO_3_ *aq*., and the desorption ratios against the adsorption amount are listed in Table 1. The CuHCF exhibited the highest adsorption and desorption amounts and desorption ratios. The desorption ratios of CoHCC and NiHCF were smaller than that of CuHCF but promising for practical use. For ZnHCF and CoHCF, NH_3_ did not desorb significantly. The worst was MnHCF, which exhibited a negative desorption amount, indicating that MnHCF adsorbed ammonium ions in the liquid during washing with sat. NH_4_HCO_3_ *aq*. The desorption amount increased as the adsorption amount increased. However, the desorption amount of CoHCC was higher than that of NiHCF, and its adsorption amount was lower than that of NiHCF.

### 2.2. Charcterization

To study the NH_3_ adsorption and desorption behavior of the PBAs, X-Ray Diffraction (XRD) and Fourier transform infrared spectroscopy (FTIR) were performed. The XRD results showed the crystal structure of each step during the NH_3_ adsorption and desorption process. Besides, it also showed the structural stability, which is an important factor for practical use and cycle stability. FTIR spectra contains information on the chemical state of NH_3_ adsorbed on PBAs and the oxidation number of iron and cobalt from the peak corresponding to the six cyano (CN) groups that coordinate to M’ in A*_y_*M[M’(CN)_6_]*_x_* [28] (pp. 110–117). NH_3_ coordination on a metal ion shifts the NH_3_ degenerate deformation to a higher wavenumber than that of the free NH_3_ molecule and is observed at 1370–1000 cm^−1^ [28] (pp. 1–6). NH_4_/NH_3_ degenerate deformation is observed at approximately 1680 cm^−1^, and NH_4_ degenerate deformation is observed at approximately 1400 cm^−1^ [28] (pp. 1–6). The CN stretch mode is observed at 2200–2000 cm^−1^, and the CN peak shifted to a lower wavenumber as M’ in A*_y_*M[M’(CN)_6_]*_x_* has been reduced [28] (pp. 110–117). The H_2_O bending mode is observed at 1630–1600 cm^−1^, without any significant difference in the peak positions between the lattice H_2_O and coordinated H_2_O [28] (pp. 57–60).

**Figure 2:**
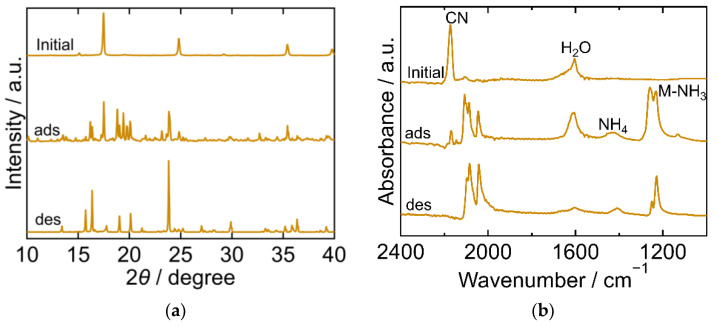
XRD patterns (**a**) and FTIR spectra (**b**) of copper hexacyanoferrate (CuHCF). “Initial” sample is literally the sample before adsorption. The samples after adsorption and desorption are denoted by “ads” and “des”, respectively. The measurements were performed at ambient temperature.

The XRD and FTIR results for CuHCF are shown in Figure 2a,b, respectively, exhibiting different characteristics at each step of the process. The XRD pattern of the initial CuHCF exhibited a cubic crystal structure (space group *Fm-3m*, *a* = 10.12 Å, PDF 01-073-9927 [29]). After adsorption, the XRD pattern became complex with peaks corresponding to before and after adsorption. After desorption, the sample showed a peak position comparable to that of copper ferrocyanide, which adsorbed NH_3_ and was washed with sat. NH_4_HCO_3_ *aq* [23]. This had an orthorhombic crystal structure (space group *Pbam*, *a* = 10.80940 Å, *b* = 3.16230 Å, *c* = 9.31820 Å, PDF00-052-0356 [29]). The FTIR spectra of the initial CuHCF exhibited peaks derived from a cyano group at 2160 cm^−1^, and H_2_O at 1605 cm^−1^. After adsorption, the CN peak was split into several peaks observed within the range of 2040–2160 cm^−1^, but at a lower wavenumber than that of the initial CuHCF. A small peak derived from ammonium ions was observed at 1440 cm^−1^ and a double peak of Cu-NH_3_ was observed at 1251 and 1227 cm^−1^. The two peaks were attributed to Cu derived from Cu-NH_3_. It is considered that there are two types of Co-NH_3_ interactions because the crystal structure became orthorhombic and, after desorption, CN peaks were observed at a lower wavenumber compared with those of the initial CuHCF and fewer peaks were observed compared with those after adsorption. The CN peak shift to a lower wavenumber after adsorption and desorption compared to the initial CuHCF indicates that the oxidation number of Fe in hexacyanoferrate decreased from three to two upon adsorption of NH_3_. The Cu-NH_3_ peak at 1251 cm^−1^ became smaller than the Cu-NH_3_ peak at 1227 cm^−1^ after desorption, whereas the NH_4_ peak remained almost the same after desorption. This indicates that the coordinated NH_3_ that resulted in the absorption at 1251 cm^−1^ was desorbed. The shape of the peak at 1606 cm^−1^ is different from that at 1605 cm^−1^ for the initial CuHCF. The H_2_O peak became smaller after desorption, indicating that lattice H_2_O or coordinated H_2_O was desorbed into sat. NH_4_HCO_3_ *aq*. The FTIR spectrum of the sample after desorption was like that of copper ferrocyanide that adsorbed NH_3_ and was washed with sat. NH_4_HCO_3_ *aq* [23]. This trend was consistent with the XRD results of the same sample.

**Figure 3:**
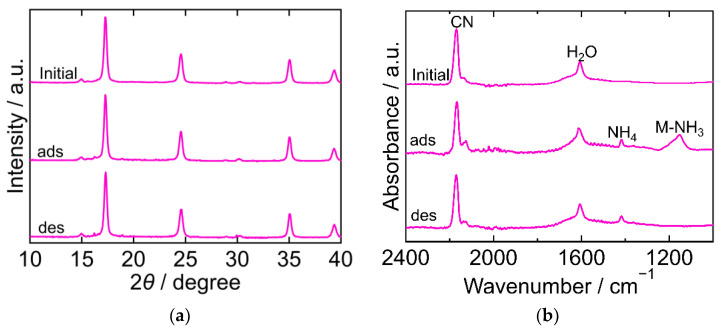
XRD patterns (**a**) and FTIR spectra (**b**) of cobalt hexacyanocobaltate (CoHCC). “Initial” sample is literally the sample before adsorption. The samples after adsorption and desorption are denoted by “ads” and “des”, respectively. The measurements were performed at ambient temperature.

The XRD and FTIR results for CoHCC are exhibited in Figure 3. The XRD patterns were almost identical at every step in the adsorption and desorption experiments and corresponded to the reported ones, indicating that the crystal structure of CoHCC was face-centered cubic (space group *Fm-3m*, *a* = 10.21 Å, PDF 01-072-1431 [29]). The XRD patterns confirm the cycling stability of CoHCC as an adsorbent. In the FTIR spectra, the peaks derived from CN and H_2_O at 2169 cm^−1^ and 1606 cm^−1^, respectively, remained unchanged during the process, indicating that the oxidation number of cobalt in the hexacyanocobaltate group and the chemical state of H_2_O in CoHCC did not change. After NH_3_ adsorption, peaks derived from NH_4_ and Co-NH_3_ were observed at 1403 cm^−1^ and 1150 cm^−1^, respectively. Part of the NH_3_ adsorbed on CoHCC is supposed to have become ammonium ions, accepting a proton from H_2_O. After desorption, the Co-NH_3_ peak was not observed, indicating that the coordinated NH_3_ desorbed into sat. NH_4_HCO_3_ *aq*.

**Figure 4:**
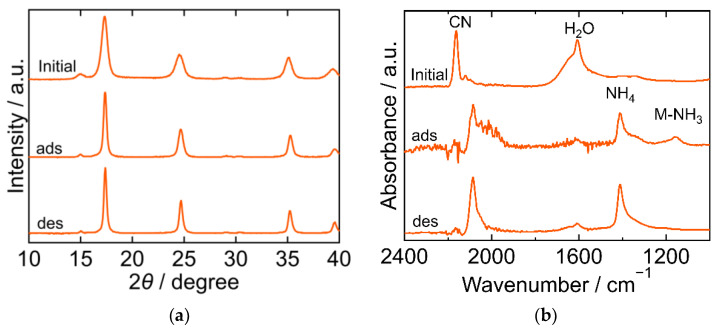
XRD patterns (**a**) and FTIR spectra (**b**) of Nickel hexacyanoferrate. “Initial” sample is literally the sample before adsorption. The samples after adsorption and desorption are denoted by “ads” and “des”, respectively. The measurements were performed at ambient temperature.

The XRD and FTIR results for the NiHCF are shown in Figure 4. The peak positions in the XRD patterns were almost identical at every step in the adsorption and desorption experiments and corresponded to the reported ones, indicating that the crystal structure of NiHCF was cubic (space group *F-43m*, *a* = 10.229 Å, PDF 00-046-0906 [29]). The unchanged peak position in the patterns at every step indicated the stability of the structure during the adsorption and desorption processes. Interestingly, the peak width of the XRD patterns became narrower after adsorption and desorption than that in the initial samples. This trend indicates that the crystallite size increased after adsorption. In the FTIR spectra, peaks derived from CN, H_2_O, NH_4_, Ni-NH_3_ were observed. The CN peak at 2162 cm^−1^ in the initial spectra shifted towards a lower wavenumber after adsorption, indicating that the oxidation number of Fe in hexacyanoferrate decreased from three to two upon adsorption of NH_3_. The H_2_O peak at 1606 cm^−1^ became smaller after adsorption and desorption than the initial peak. NH_4_ and Ni-NH_3_ peaks were observed after adsorption at 1412 cm^−1^ and 1159 cm^−1^, respectively. The Ni-NH_3_ peak became undetectable after desorption, while the NH_4_ peak remained.

**Figure 5:**
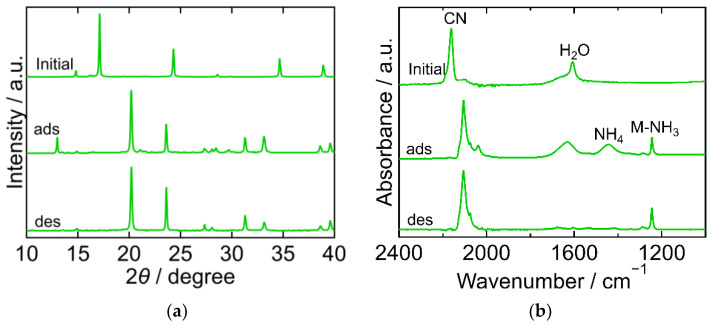
XRD patterns (**a**) and FTIR spectra (**b**) of zinc hexacyanoferrate (ZnHCF). “Initial” sample is literally the sample before adsorption. The samples after adsorption and desorption are denoted by “ads” and “des”, respectively. The measurements were performed at ambient temperature.

The XRD and FTIR results for ZnHCF are shown in Figure 5. The XRD pattern of the initial ZnHCF exhibited a cubic framework (space group *Fm-3m*, a = 10.3392 Å, PDF 01-076-5123) [29]. After adsorption, the XRD pattern was clearly different from the initial one and became comparable to that of the hexagonal framework of Zn^II^[Fe^III^(CN)_6_]_0.5_ (space group *P-3*, *a* = 7.5803 Å, *c* = 5.7363 Å, *a*/*b* = 1, *c*/*b* = 0.75674, PDF 01-077-6230 [29]). After desorption, the XRD pattern was almost the same as that after adsorption, but a peak at 2*θ* = 13° was not observed. The FTIR spectra exhibited peaks corresponding to CN, H_2_O, NH_4_, Zn-NH_3_. The CN peak at 2162 cm^−1^ in the initial spectra shifted to a lower wavenumber, indicating that Fe was reduced. The NH_4_ peak and Zn-NH_3_ peak appeared at 1442 cm^−1^ and 1445 cm^−1^, respectively, after adsorption, and only the Zn-NH_3_ peak remained after desorption. This demonstrates that the coordinated NH_3_ desorbed into sat. NH_4_HCO_3_ *aq*. The H_2_O peak at 1607 cm^−1^ in the initial spectra became relatively symmetric and its position shifted to 1632 cm^−1^. These changes might result from the degenerated deformation of NH_3_/NH_4_ or a change in the chemical state of H_2_O in ZnHCF. No peak was observed in the range 1600–1632 cm^−1^ after desorption, indicating that lattice H_2_O or coordinated H_2_O was desorbed into sat. NH_4_HCO_3_ *aq*.

**Figure 6:**
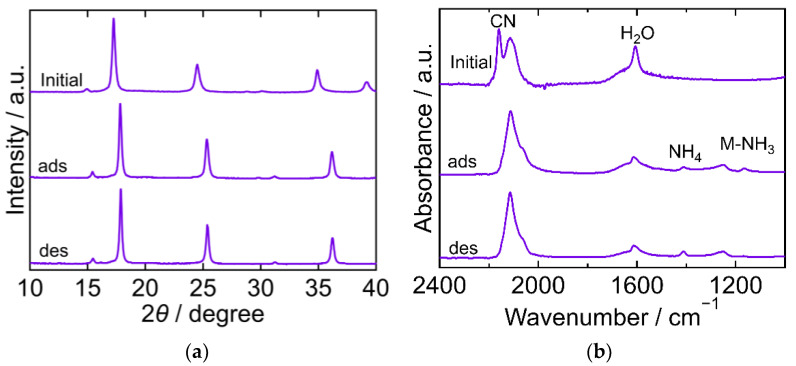
XRD patterns (**a**) and FTIR spectra (**b**) of cobalt hexacyanoferrate (CoHCF). “Initial” sample is literally the sample before adsorption. The samples after adsorption and desorption are denoted by “ads” and “des”, respectively. The measurements were performed at ambient temperature.

The XRD and FTIR results for CoHCF are shown in Figure 6. The XRD pattern of the initial CoHCF exhibited a cubic framework (space group *Fm-3m*, *a* = 10.2666 Å, PDF 01-085-2546 [29]). After adsorption, the XRD peak position shifted to a higher 2*θ* value and corresponded to that of an orthorhombic framework (space group *Pmn21*, *a* = 9.933 Å, *b* = 6.989 Å, *c* = 7.066 Å, *a*/*b* = 1.42123, *c*/*b* = 1.01102, PDF 01-075-9531 [29]). The peak position after desorption was almost the same as that after adsorption, demonstrating that the structure did not change owing to desorption. The FTIR spectra exhibited peaks corresponding to CN, H_2_O, NH_4_, and Co-NH_3_. CN peaks were observed at 2159 cm^−1^ and 2114 cm^−1^ suggesting the presence of divalent Fe in the initial CoHCF, although it was synthesized from a chemical with trivalent Fe. All the Fe appeared to be divalent both after adsorption and desorption. The CN peak was observed at 2112 cm^−1^. The H_2_O peak was observed at 1607 cm^−1^ in the initial spectra and remained at the same position both after adsorption and desorption. The NH_4_ peak appeared after adsorption and remained even after desorption. The two peaks that appeared at 1250 cm^−1^ and 1165 cm^−1^ were supposedly derived from Co-NH_3_. The peak at 1165 cm^−1^ was not observed after desorption. These results indicate that there are two types of Co-NH_3_ interactions because the crystal structure became orthorhombic. The NH_3_ from the Co-NH_3_ interaction that yielded the peak at 1165 cm^−1^ desorbed.

**Figure 7:**
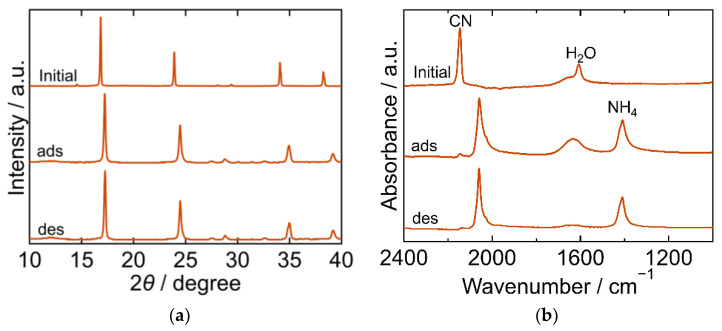
XRD patterns (**a**) and FTIR spectra (**b**) of manganese hexacyanoferrate (MnHCF). “Initial” sample is literally the sample before adsorption. The samples after adsorption and desorption are denoted by “ads” and “des”, respectively. The measurements were performed at ambient temperature.

The XRD and FTIR results for the MnHCF are shown in Figure 7. The XRD pattern of the initial MnHCF exhibited a cubic framework (space group *Fm-3m*, *a* = 10.488 Å, PDF 01-074-7327 [29]). After adsorption, the XRD peak shifted to a higher 2*θ*. The peak position after desorption was almost the same as that after adsorption, demonstrating that the structure did not change owing to desorption. The FTIR spectra exhibited peaks derived from CN, H_2_O, and NH_4_. The CN peak at 2144 cm^−1^ in the initial spectra shifted to a lower wavenumber after adsorption, and its position remained the same after desorption, indicating that Fe was reduced on adsorption. The H_2_O peak became symmetric after adsorption, and its absorbence decreased after desorption due to H_2_O desorption into sat. NH_4_HCO_3_ *aq*. The Mn-NH_3_ peak was not observed at any step during the process, whereas the NH_4_ peak at 1403 cm^−1^ appeared after adsorption and remained even after desorption. These results indicated that the adsorbed NH_3_ did not yield Mn-NH_3_ and that ammonium ions were not desorbed into sat. NH_4_HCO_3_ *aq*. This is consistent with the result that the desorption of the NH_3_ from MnHCF was not detected.

### 2.3. The Crystal Structure and Chemical State during the Process

Through the characterization of six kinds of PBAs at each step in the adsorption and desorption processes, changes in the crystal structure and chemical state were identified. The XRD patterns showed that the structural stability of CoHCC and NiHCF was high. The structures of CuHCF and ZnHCF changed drastically upon adsorption, possibly because CuHCF and ZnHCF prefer tetrahedral coordination. FTIR results indicated that NH_3_ adsorbed on PBA can take two states as explained previously [13]. NH_3_ is adsorbed on M in PBA (A*_y_*M[M’(CN)_6_]*_x_*) as follows:

(1) $$\mathrm{PBA}\cdot nH_{2}O\;+m{\mathrm{NH}}_{3}\;\to \;\mathrm{PBA}\cdot \left(n-m\right)H_{2}O\cdot m{\mathrm{NH}}_{3}+mH_{2}O.$$

Then, part of the adsorbed NH_3_ reacts with H_2_O in PBA as follows [13]:

(2) $$\mathrm{PBA}\cdot \left(n-m\right)\;\left(H_{2}O\right)\cdot m{\mathrm{NH}}_{3}\;\to \;{\left({\mathrm{NH}}_{4}\right)}_{l}\mathrm{PBA}\cdot l\mathrm{OH}\cdot \left(n-m-l\right)H_{2}O\cdot \left(m-l\right){\mathrm{NH}}_{3}$$

There was a tendency for the coordinated NH_3_ observed as an M-NH_3_ peak at 1370–1000 cm^−1^ in the FTIR spectra to be desorbed into sat. NH_4_HCO_3_ *aq*, whereas ammonium ions observed as NH_4_ peaks at approximately 1400 cm^−1^ usually remained in PBA.

(3) $${\left({\mathrm{NH}}_{4}\right)}_{l}\mathrm{PBA}\cdot l\mathrm{OH}\cdot \left(n-m-l\right)H_{2}O\cdot \left(m-l\right){\mathrm{NH}}_{3}\;\to \;{\left({\mathrm{NH}}_{4}\right)}_{l}\mathrm{PBA}\cdot l\mathrm{OH}\cdot \left(n-m-l\right)H_{2}O\cdot \left(m-l-j\right){\mathrm{NH}}_{3}+j{\mathrm{NH}}_{3}$$

Coordinated NH_3_ refers to NH_3_ molecules adsorbed on vacancy sites, while ammonium ions are trapped on interstitial sites [13]. The results showed that NH_3_ molecules adsorbed on vacancy sites were more likely to be desorbed into sat. NH_4_HCO_3_ *aq,* whereas ammonium ions in the interstitial sites were not prone to desorption. ZnHCF was an exceptional case in which the ammonium ions in the interstitial sites were desorbed and coordinated NH_3_ at the vacancy sites remained as follows:

(4) $${\left({\mathrm{NH}}_{4}\right)}_{l}\mathrm{PBA}\cdot l\mathrm{OH}\cdot \left(n-m-l\right)H_{2}O\cdot \left(m-l\right){\mathrm{NH}}_{3}\to \;\mathrm{PBA}\cdot \left(n-m-l\right)H_{2}O\cdot \left(m-l\right){\mathrm{NH}}_{3}+l{\mathrm{NH}}_{3}+lH_{2}O.$$

One possible explanation for this is that the crystal structure of ZnHCF became a hexagonal framework after adsorption.

### 2.4. The Desorption/Adsorption Ratio

**Figure 8:**
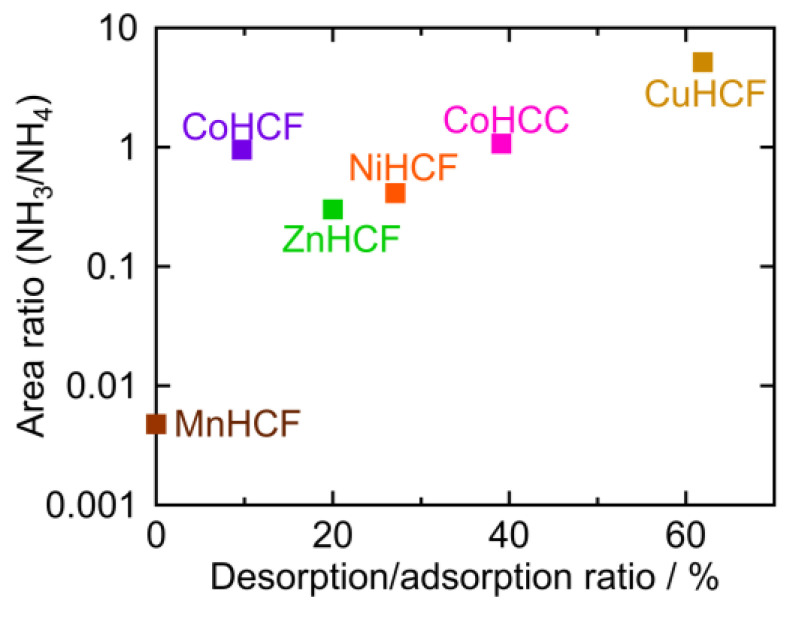
The area ratio of the M-NH_3_ and NH_4_ peak as observed in the FTIR spectra.

The peak area ratios of NH_4_ and M-NH_3_ are plotted against the desorption ratio in Table 1 (Figure 8). To calculate the peak area ratio, the baseline was first determined as the line through the two points in the raw data at 1769 cm^−1^ and 1016 cm ^−1^. The baseline was subtracted from the raw data. The areas of the NH_4_ peak around 1400 cm^−1^ and M-NH_3_ peak at 1370–1000 cm^−1^ were calculated, and the ratio of the two peaks (NH_3_/NH_4_) was derived. This area ratio was plotted on a logarithmic scale because it should be proportional to the equilibrium constant. CuHCF had the highest desorption/adsorption ratio and the highest NH_3_/NH_4_ peak area ratio, while MnHCF had the lowest desorption/adsorption and the lowest NH_3_/NH_4_ peak area ratio. For other PBAs, the desorption/adsorption ratio was higher if the NH_3_/NH_4_ peak area ratio was higher, except in the case of CoHCF. Interestingly, PBA with a higher NH_3_/NH_4_ ratio, released NH_3_ into sat. NH_4_HCO_3_ *aq*. A higher NH_3_/NH_4_ ratio indicated a higher affinity for NH_3_ at the vacancy sites.

**Figure 9:**
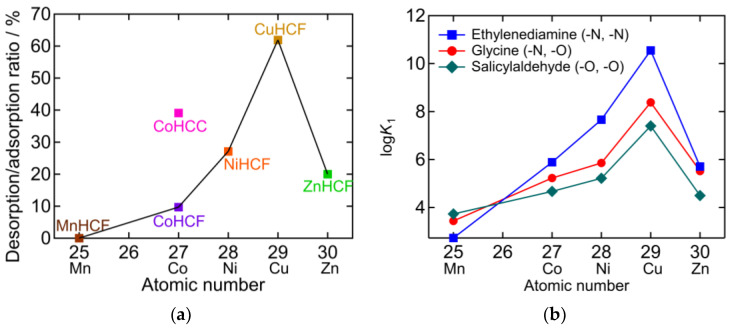
(**a**) The desorption/adsorption ratio plotted against atomic number of M in PBA (A*_y_*M[M’(CN)_6_]*_x_*). Lines connect hexacyanoferrate PBAs showing the same order as the Irving-Williams series [30]. (**b**) Stability constants of divalent transition metal chelate complex [31,32,33,34,35,36].

In Figure 9a, the desorption/adsorption ratio is plotted against the atomic number M in PBA (A*_y_*M[M’(CN)_6_]*_x_*). The desorption/adsorption ratio of hexacyanoferrate (HCF) PBAs obeys the Irving-Williams order [30]. This indicates that there is a relationship between the NH_3_ adsorption/desorption behavior and the stability of the metal complexes. The higher desorption/adsorption ratio of CoHCC compared to the HCF series suggests that hexacyanocobaltate PBAs may exhibit higher desorption/adsorption ratios. Figure 9b shows the stability constants (*K*_1_) of the divalent transition metal chelate complex, which also obey the Irving-Williams order [31,32,33,34,35,36]. The chelates are ethylenediamine (en), glycine (gly), and salicylaldehyde (sald), whose coordination elements are double nitrogen (N), N, oxygen (O), and double O, respectively. *K*_1_ becomes larger as N in the chelate increases in the cases of Co^2+^, Ni^2+^, Cu^2+^, and Zn^2+^. This is consistent with Equation (1), which occurred for CoHCF, CoHCC, NiHCF, CuHCF, and ZnHCF, as shown in Figure 2b, Figure 3b, Figure 4b, Figure 5b and Figure 6b, respectively. In contrast, in the case of Mn, *K*_1_ increased as O in the chelate increased. This indicates that Mn^2+^ has a higher affinity for O than for N. This is consistent with the result that, after adsorption, only the NH_4_ peak was observed with MnHCF, as shown in Figure 7b. Except for MnHCF, it is favorable for NH_3_ to be adsorbed on the vacancy sites instead of coordinating with H_2_O in the gaseous phase. In sat. NH_4_HCO_3_ *aq*, the liquid phase is more favorable for NH_3_ than being adsorbed at the vacancy site. Therefore, desorption in sat. NH_4_HCO_3_ *aq* is not necessarily an exchange between NH_3_ and H_2_O because the H_2_O peak was unchanged or not observed in the FTIR spectra after desorption.

## 3. Materials and Methods

### 3.1. Materials

The chemicals used for synthesis were purchased from Wako Pure Chemical Industries, Ltd. (Osaka, Japan) at the special grade. First grade ammonium hydrogen carbonate for NH_3_ desorption was purchased from Wako Pure Chemical Industries, Ltd.. Sodium hydrogen sulfate was purchased from Wako Pure Chemical Industries, Ltd., at a special grade. All chemicals were used without further purification.

### 3.2. Synthesis of PBAs

The chemicals used for the synthesis were dissolved in ultra-pure water in a centrifuge tube. The same volume of the solutions were mixed for the synthesis. CuHCF was synthesized by mixing solutions of 0.6 mol L^−1^ copper sulfate, and 0.2 mol L^−1^ potassium ferricyanide. CoHCC was synthesized from solutions of 0.6 mol L^−1^ of cobalt(II) chloride and 0.2 mol L^−1^ of potassium hexacyanocobaltate(III). NiHCF was obtained by mixing solutions of 0.6 mol L^−1^ of nickel(II) nitrate and 0.2 mol L^−1^ of potassium ferricyanide. ZnHCF was synthesized from 0.6 mol L^−1^ of zinc sulfate and 0.2 mol L^−1^ of potassium ferricyanide. CoHCF was obtained from solutions of 0.6 mol L^−1^ of cobalt(II) chloride and 0.4 mol L^−1^ of potassium ferricyanide. MnHCF was synthesized from solutions of 0.3 mol L^−1^ of manganese(II) chloride and 0.3 mol L^−1^ of potassium ferricyanide. For the PBA-forming reaction, the samples were shaken overnight at 1000 rpm using a SI-300C shaker (AS-ONE Corp., Osaka, Japan). The PBA dispersed in the solution was precipitated by centrifuging the PBA dispersion at 16,000× *g* for 10 min using a centrifuge (3–30 K, Sigma Laborzentrifugen GmbH, Osterode am Harz, Germany). To rinse the salt impurity from PBA, the supernatant was removed and the same amount of ultrapure water was added again. This process was repeated six times. After the rinsing, the purities of PBAs were higher than 99%, which was confirmed using scanning electron microscope-energy dispersive x-ray spectrometry (SEM-EDX, TM4000Plus, Hitachi High-Tech Corp., Tokyo, Japan) assuming that the possible impurity was a salt. The yield of the synthesized CuHCF, CoHCC, NiHCF, ZnHCF, CoHCF and MnHCF was, respectively 55%, 57%, 64%, 48%, 61% and 44%. After rinsing, the precipitate was dried overnight under vacuum, and subsequently, ground to a powder form.

### 3.3. The Experimantal Scheme of the NH_3_ Adsorption and Desorption

**Figure 10:**
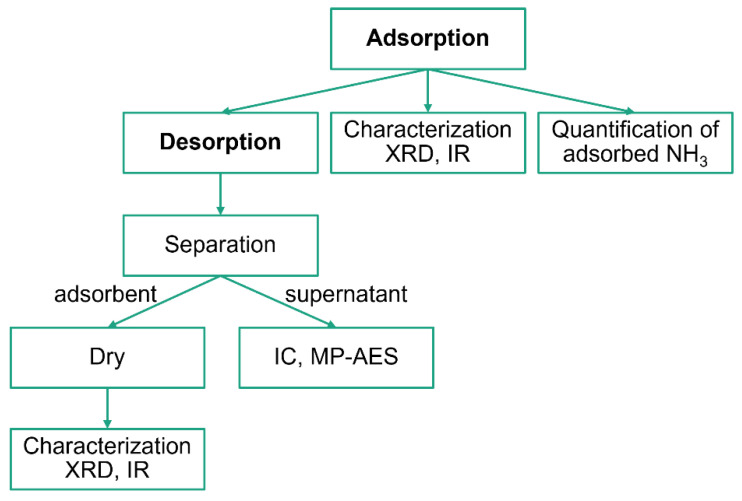
The experimental scheme.

The experimental scheme is illustrated in Figure 10. The main events were NH_3_ adsorption and desorption, whereas the other events were analytical experiments. The entire process was conducted at ambient temperature.

**Figure 11:**
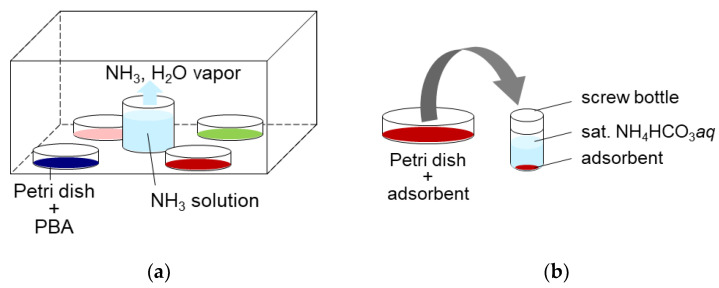
(**a**) The adsorption method. (**b**) The desorption method.

For NH_3_ adsorption, approximately 1 g of each PBA powder was placed in a sealed space with 100 mL of 2.8% ammonia solution, and left for 3.7 days, as shown in Figure 11a. This yielded NH_3_ and water vapor. The gaseous NH_3_ concentration was measured several times using gas detection tubes (3HM, GASTEC Co., Ayase, Japan) to confirm whether the system reached equilibrium. The NH_3_ and H_2_O concentrations were 17,600 ppmv and 23,000 ppmv, respectively. After adsorption, PBA powder was used for desorption, characterization, and quantification of adsorbed NH_3_.

For the desorption of NH_3_ adsorbed on PBAs, approximately 300 mg of PBA powder was placed in a 6 mL screw bottle and sat. NH_4_HCO_3_ *aq* was added at a liquid/solid ratio of 5 (wt./wt.), as shown in Figure 11a. The six screw bottles were shaken for >48 h at 300 rpm using a shaker (SI-300C). The samples were then left on a table until the PBA powder precipitated, and the supernatant became clear. An aliquot of the supernatant was collected and diluted 10,000 times with ultrapure water. The diluted solutions were filtered to remove the remaining PBA powder using a syringe and a PTFE filter with a pore size 0.45 mm. They were then subjected to ion chromatography (IC, Metrohm, Herisau, Switzerland) to measure the concentration of NH_4_^+^ and microwave plasma atomic emission spectrometry (MP-AES, Agilent Technologies Japan, Ltd., Hachioji, Japan) to measure dissolved metallic ions from PBA. The supernatant was removed using a pipette, and the precipitate was dried on a filter paper for XRD and FTIR measurements.

The adsorbed NH_3_ was quantified by acid-washing the adsorbent. After NH_3_ adsorption, the PBAs were washed with 30 mmol L^−1^ sodium hydrogen sulfate solution (NaHSO_4_ *aq*). Approximately 20 mg of PBA was put into a 50 mL centrifuge tube, and NaHSO_4_ *aq* was added at a liquid/solid ratio of 500 (wt./wt.). The PBA in NaHSO_4_ *aq* was shaken for more than 24 h at 600 rpm using a shaker (SI-300C). The liquid and the adsorbent were separated by centrifuging at 16,000× *g* for 5 min. The supernatant was diluted 1000 times with ultrapure water and processed for IC measurements to measure the concentration of NH_4_^+^.

The PBAs were characterized at each step of the XRD and ATR-FTIR experiments. PBA powder was ground using an agate mortar and pestle for the measurements. XRD measurements to examine the crystal structure of the PBAs were conducted using an X-ray diffractometer (D8 ADVANCE, Bruker Corp., Billerica, MA, USA) with Cu Kα radiation (λ = 0.154 nm) at 40 kV and 40 mA at ambient temperature. The PBA samples for XRD measurements were evenly applied to a reflection-free sample holder. ATR-FTIR spectra were obtained using a diamond ATR unit (Nicolet iS5; Thermo Fisher Scientific Inc., Waltham, MA, USA).

## 4. Conclusions

In this study, we tested six types of PBAs to investigate the NH_3_ adsorption/desorption behavior when the desorption method was washing with sat. NH_4_HCO_3_ *aq*. Sat. NH_4_HCO_3_ *aq* was used as the washing liquid to recover NH_3_ as solid NH_4_HCO_3_ by introducing CO_2_ into the washing liquid after desorption. In the experiment, the PBAs were left in a sealed space with an NH_3_ solution for adsorption. Then, the PBAs were washed with sat. NH_4_HCO_3_ *aq* for desorption. The amounts of adsorbed and desorbed NH_3_ were quantified using IC. The PBAs were analyzed by XRD and FTIR measurements.

The amounts of adsorption and desorption and the desorption/adsorption ratio were the highest for CuHCF. The XRD results showed that the structural stability is higher when the metal ions in PBAs are hexacoordinated rather than tetracoordinated. For practical use as adsorbents, PBAs containing hexacoordinated metal ions are expected to have cyclic stability. Further studies on the adsorption/desorption properties during the second and third cycles are required. The FTIR results indicated that NH_3_ adsorbed on the vacancy sites could be desorbed by washing with sat. NH_4_HCO_3_ *aq* compared with NH_4_^+^ in the interstitial sites. The relationship between the desorption/adsorption ratio and the adsorption sites was discussed based on the peak area ratio of the NH_3_ peak and NH_4_ peak observed in the FTIR spectra. The desorption/adsorption ratio increased with the NH_3_/NH_4_ peak area ratio. Furthermore, the desorption/adsorption ratio followed the Irving-Williams order. These results indicate the NH_3_ adsorption/desorption behavior is related to the stability of the metal complexes. Hexacyanocobaltate PBAs are expected to have higher desorption/adsorption ratio compared to ferricyanide PBAs. We have reported that the desorption amount of NH_3_ from activated carbon, zeolite, and an ion exchange resin were less than 0.5 mmol when 1 g of adsorbents were used for adsorption in 60% NH_3_ gas and desorption by washing with sat. NH_4_HCO_3_ *aq* [23]. In this study, we have demonstrated that the NH_3_ adsorption/desorption behavior can be improved by changing metals in PBAs, showing that PBAs are promising NH_3_ adsorbents for ammonia recovery as an NH_4_HCO_3_ solid compared to activated carbon, zeolite, and ion exchange resins. The results obtained in this study will contribute to the development of adsorbents for NH_3_ recovery as reusable solids.

## Figures and Tables

**Table 1:** The adsorption and desorption amount of NH_3_ and the ratio of desorption.

PBA	Adsorption Amount mmol/g	Desorption Amount mmol/g	Desorption Ratio %
CuHCF	13.2	8.19	61.9
CoHCC	4.87	1.90	39.1
NiHCF	5.48	1.49	27.1
ZnHCF	2.66	0.53	20.0
CoHCF	1.47	0.14	9.7
MnHCF	4.57	−0.63	−13.9

## Data Availability

The data presented in this study are available on request from the corresponding author.

## Sample Availability

Samples of the compounds are not available from the authors.
